# The Use of *Perovskia abrotanoides* Extract in Ameliorating Heat Stress-Induced Oxidative Damage and Improving Growth Efficiency in Carp Juveniles (*Cyprinus carpio*)

**DOI:** 10.1155/2024/5526562

**Published:** 2024-06-25

**Authors:** Hossein Adineh, Saeed Zahedi, Morteza Yousefi, Zeynab Sedaghat, Sevdan Yilmaz, Ebrahim Gholamalipour Alamdari, Mohammad Farhangi

**Affiliations:** ^1^ Department of Fisheries Faculty of Agriculture and Natural Resources Gonbad Kavous University, Gonbad Kavous, Golestan, Iran; ^2^ Department of Fisheries Faculty of Natural Resources and Environment Ferdowsi University of Mashhad, Mashhad, Iran; ^3^ Department of Veterinary Medicine RUDN University, 6 Miklukho-Maklaya St, Moscow 117198, Russia; ^4^ Department of Aquaculture Faculty of Marine Sciences and Technology Canakkale Onsekiz Mart University, Canakkale 17100, Türkiye; ^5^ Department of Plant Production Faculty of Agriculture and Natural Resources Gonbad Kavous University, Gonbad Kavous, Golestan, Iran

## Abstract

Herbal extracts have been successfully used as feed additives in fish culture with attractive growth-promoting, immunostimulant, antimicrobial, and antioxidant properties for several fish and shellfish species. Therefore, we have designed a feeding trial to assess the impacts of dietary incorporation of *Perovskia abrotanoides* extract (PAE) on common carp (*Cyprinus carpio*). For this purpose, five isonitrogenous (35% protein) and isocaloric (~4,000 kcal/kg) diets have been supplied by supplementing PAE at the varying inclusion levels as 0.0%, 0.25%, 0.5%, 1.0%, and 2.0% diets, and growth performance and feed utilization, digestive enzyme activities, serum biochemical variables, antioxidant responses, and immunological factors were studied. The experiment continued for 60 days. At the termination of the experiment, the mean final weight, weight gain percentage (WG%), feed conversion rate (FCR), and specific growth rate (SGR) have been improved significantly in all fish groups fed PAE-based diets with regard to those fed the reference diets. A second-order polynomial regression equations indicate that the optimum dietary supplementation level of PAE in fish diets was ~1%. Serum cortisol, glucose, triglyceride, cholesterol, and malondialdehyde levels as well as catalase, alanine aminotransferase, and aspartate aminotransferase activities were significantly decreased generally in all PAE-supplemented groups compared to the control groups before and/or after high-temperature stress (32°C). Moreover, serum total protein, albumin, and total immunoglobulin levels as well as ACH50, lysozyme, superoxide dismutase, and glutathione peroxidase activities were increased before and/or after high-temperature stress (32°C). In conclusion, the results showed, for the first time, that dietary supplementation with ~1% PAE can improve growth performance, stimulated the digestive enzymes, and enchanced antioxidant status as well as immune parameters and prevented high-temperature stress of common carp.

## 1. Introduction

With the global population steadily expanding, the imperative to enhance food production becomes increasingly urgent. The alteration in human consumption trends, encompassing a movement toward aquatic sources of protein, especially derived from aquaculture, emerges as a wholesome and ecologically viable substitute for diets centered around meat [1, 2]. Aquaculture is a significant economic sector that offers humans a useful and necessary protein supply [3, 4]. Nowadays, aquaculture provides over 50% of the global fish supply intended for human consumption [5]. The cyprinid fish family has gained high economic importance as it contributes more than 20 million metric tons of fish to world fish production, constituting approximately 40% of the total output from aquaculture worldwide and encompassing 70% of freshwater aquaculture yield [5]. Carps are an environmentally friendly alternative to other major aquaculture species such as salmon and shrimp. They are omnivorous/filter fish and require less fish meal and fish oil when farmed. Common carp (*Cyprinus carpio*) is the main member of the cyprinid fish family, which is farmed in more than 100 countries. It accounts for up to 10% (over 3 million tons) of the global annual freshwater aquaculture production [6]. The common carp ranks fourth among the most common cultivated fish species in the world and is highly valued in many countries of the Middle East, including Iran [7]. In its natural habitat, this species is found in the Caspian Sea, where it is actively farmed in most freshwater basins, including aquaculture farms located in the southern part of the Caspian Sea [8].

Fish have evolved to survive in a specific range of environmental variations, and anything outside of that ranges threatens their physiology and health [9, 10]. Also, intensive aquaculture systems may cause some environmental factor changes that facilitate the occurrence of disease outbreaks due to imposed stress conditions and subsequent fish immunosuppression [11]. Hence, the management of environmental factors holds paramount importance, with temperature emerging as the most crucial factor among them. It influences all aspects of aquatic animal life such as their survival, metabolism, feeding habits, growth performance, and disease resistance. Severe heat stress depresses the growth, physiological function, feed efficiency, oxidative status, and immune response in the organs of aquatic animal [12] and, subsequently, reduced the fish ability to defend against infectious diseases [13]. In this regard, improving the immune system of fish during heat stress is of great importance for fish farmers.

Nowadays, the use of medicinal plants is recommended for stress relief in aquaculture. The *Perovskia* genus, Labiatae family, ranks among the frequently utilized medicinal plants and comprises seven distinct species. From this family, three species including *P. abrotanoides*, *P. atriplicifolia*, and *P. artemisoides* are observed in the nature of Iran [14]. *P. abrotanoides* represents a perennial herbaceous plant that thrives in various geographical areas such as Iran, Afghanistan, Turkmenistan, and Pakistan, known by various names including Brazambal, Domou, and Gevereh. Some pharmacological actions of the plant have been verified including leishmanicidal, antiplasmodial, and antinociceptive and antiinflammatory activities [15, 16, 17]. Employing *P. abrotanoides* oil in the treatment of secondary infections associated with leishmaniasis, particularly in relation to *S. aureus*, holds potential advantages [18]. Tanshinone, a diterpene molecule found in *P. abrotanoides* roots, has been demonstrated to have a variety of biological actions, including antibacterial, antioxidant, and anticancer characteristics [14, 16]. Local communities use the herb to treat typhoid fever, migraine, arteriosclerosis, vomiting, gonorrhea, cough, toothache, and cardiovascular and liver disease [19, 20, 21]. It has antiseptic, sedative, analgesic, and cooling effect [15, 22]. GC and GC-MS were used to examine the essential oil extracted from the flowering aerial portions of *P. abrotanoides*. There were 21 components were found. The composition of the essential oil includes mainly camphor (34.1%), 1.8-cineole (18.0%), b-caryophyllene (8.2%), and a-humulene (8.2%) [23].

Herbal feed supplements are renowned for their wide range of beneficial properties, such as antistress and antioxidant effects, immune-boosting capabilities, and hepatoprotective properties. Therefore, nutritional herbal supplements are a promising option for mitigating the heat stress of fish in aquaculture conditions [24]. For example, the previous study has shown that hyssop (*Hyssopus officinalis*) extract inclusion into the diet improves the physiological and antioxidant responses of young rainbow trout (*Oncorhynchus mykiss*) exposed to thermal stress [25]. However, as far as literature survey could ascertain, the protective effect of *P. abrotanoides* extract (PAE) in heat stressed carp juveniles has yet to be determined. Also, the effect of *P. abrotanoides* extract on growth efficiency in carp juveniles (*C. carpio*) has not been documented before in prior records. Hence, the main goal of this research was to examine the impact of orally administering *P. abrotanoides* extract on the productivity, biochemical, and immune characteristics of carp.

## 2. Materials and Methods

### 2.1. Plant Collection and Extraction of Phenolic Compounds

Samples of *Proviskia abrotanoides* were collected in the Vamnan region located in the Azadshahr county, Golestan province, in northern Iran, at the flowering period. The collected plant sample of *P. abrotanoides* was botanically authenticated by the colored flora of Iran (code no. GKU/803894). The collected samples were divided into different parts of the root, stem, leaf, and flower. The samples were washed with distilled water and then dried in the shade for 3 days and then dried in an oven at 40°C. The samples were powdered with a grinder and passed through an eight-mesh sieve and stored in plastic bags at a temperature of −20°C before beginning the experiment.

Hot extraction method was used to extract the phenolic compounds. The methanolic extract of *P. abrotanoides* has the maximum ability to extract the phenolic compounds as compared to other solvents [26]. Thus, 0.1 g of the powdered samples of the entire *P. abrotanoides* organ was weighed with a digital scale, and then 20 mL of 80% methanol was added to it; the resulting mixture was placed in a shaker for 6 hr. Then it was placed in a water bath with a temperature of 50°C for 2 hr. After this period, the obtained extracts were filtered through filter paper and brought to final volume of 25 mL with 80% methanol solvent. The profile of the studied aerial plant part of *P. abrotanoides* in the studied place was previously reported by Alamdari et al. [27] according to the Table 1 (GC-MS analysis).

### 2.2. Diet Preparation and Extract Supplementation

The main dietary formula (control) was prepared by combining the appropriate ingredients, including fish meal, meat meal, soy meal, wheat meal, fish oil, soybean oil, corn flour, lysine, methionine, and vitamins and mineral premix, in the required proportions. Then, it moistened by adding sterilized water. The prepared dough was divided into five equal parts. The control and experimental diets were prepared in the same way by adding 0%, 0.25%, 0.5%, 1%, and 2% of *P. abrotanoides* extract of dough that thoroughly mixed (Table 2). The dough that was prepared underwent extrusion using a meat grinder die. Subsequently, the resulting strands were crushed to achieve the desired size and then air-dried at room temperature. The juveniles were fed with the control and experimental diets at 2.5% of biomass for 60 days [28].

### 2.3. Fish and Experimental Conditions

The research was conducted at the Fisheries Laboratory of Gonbad Kavous University in Golestan, Iran, in full compliance with the standards and guidelines set forth by the Animal Care Committee of our university. Carp juveniles (*C. carpio*) were acclimated for 14 days in our laboratory conditions. During acclimation period, they fed 2.5% biomass three times a day (at 09:00, 13:00, and 17:00) with a commercial diet without supplementation. Four hundred fifty carp juveniles (initial weight 17.79 ± 0.86 gr) were placed in a randomized manner across 15 tanks (60 L) in five treatments (with three replicates). Water temperature, dissolved oxygen, pH, and total ammonia concentration were 26.1 ± 0.29°C, 6.76 ± 0.39 mg/L, 7.7 ± 0.21, and 0.10 ± 0.022 mg/L, respectively.

#### 2.3.1. High-Temperature Stress

At the experiment's conclusion, a batch of 10 fish per tank (totaling 30 fish per treatment) was subjected to elevated temperatures (32°C) in their own tanks for 48 hr. It should be mentioned that water temperature gradually increased as 1°C/30 min by help of aquarium heaters [29]. During this high-temperature stress period, the fish fasted completely, and the tanks were aerated continuously [30]. After 48 hr, blood samples were drawn, and the biochemical stress-related factors (glucose and cortisol) were measured.

#### 2.3.2. Sample Collection

At the experiment's conclusion, three fish were removed from each tank and then anesthetized in a clove powder bath (200 ppm) [31]. Blood samples were taken from the caudal vessel of each fish using nonheparinized syringes. In each repetition, the blood of three fish was equally mixed with each other (a total of three blood samples from each treatment). Blood was centrifuged at 5,000 rpm for 10 min. The obtained serum was preserved at a temperature of −80°C and then used for subsequent biochemical assays.

### 2.4. Analysis

#### 2.4.1. Growth Performance and Nutrient Efficiency Indices

At the conclusion of the 60-day experimental period, all fish were weighted (BW) by digital scale, and their lengths (TL) were measured by ruler. The fish growth and nutrient efficiency indices were calculated as follows [32]:

(1) $$\begin{array}{c} \text{Survival rate }\left(\%\right)=\frac{\text{final number of fish}}{\text{initial number of fish}}\times 100 \end{array}$$

(2) $$\begin{array}{c} \text{Weight gain rate }\left(\text{WGR},\%\right)=\frac{\left(\text{final weight}-\text{initial weight}\right)}{\text{initial weight}} \end{array}$$

(3) $$\begin{array}{c} \text{Specific growth rate }\left(\text{SGR},\%\;day^{-1}\right)=\frac{\left(\text{ln final weight}-\text{ln initial weight}\right)}{\text{days}}\times 100 \end{array}$$

(4) $$\begin{array}{c} \text{Feed conversion ratio }\left(\text{FCR},gg^{-1}\right)=\frac{\text{dry feed intake}}{\left(\text{final weight}-\text{initial weight}\right)} \end{array}$$

(5) $$\begin{array}{c} \text{Food conversion efficiency }\left(\text{FCE},\%\right)=\left[\frac{\left(\text{final weight}-\text{initial weight}\right)}{\text{ dry feed intake}}\right]\times 100 \end{array}$$

#### 2.4.2. Digestive Enzyme Activity Assays

At the conclusion of the 60-day experimental period, three fish from each tank were captured. Then, the fish belly was disinfected with alcohol. The intestine completely removed from the body, washed with physiological serum, and homogenized. After that, the homogenate was centrifuged (2,500x*g*, 4°C, 20 min), and the supernatant was snap frozen in liquid nitrogen [31]. The samples were weighed with a digital scale with an accuracy of 0.001 g and then weighed according to the weight-to-volume ratio (1–9) with a buffer solution (100 mM Tris-Hcl, 0.1 mM EDTA, 0.1% Triton at pH 7.8) was homogenized [33, 34].

Amylase activity was evaluated following the procedure outlined by [35], utilizing a substrate of 0.3% soluble starch that was dissolved in NaH_2_PO_4_ buffer (pH 7.4). Lipase activity was assessed over a 15-min period at 30°C using p-nitrophenyl myristate as the substrate, which was dissolved in 0.25 M Tris-HCl (pH 9.0) as per the method described by Iijima et al. [36]. Protease activity was determined at 25°C, employing a substrate of 1% (w/v) casein sourced from Sigma, USA, in 0.2 M phosphate buffer with a pH of 7.0, following the methodology of Walter [37].

#### 2.4.3. Biochemical and Immune Parameters

Using commercial kits (Pars Azmun, Karaj, Iran) following the manufacturer's instructions, calorimetric measurements were performed to determine serum total protein and albumin concentrations. Globulin concentrations were determined by subtracting the albumin content from the overall serum protein. Serum cholesterol and triglycerides were analyzed using commercial kits (ZiestChem Diagnostics, Tehran, Iran) using the method proposed by the manufacturer.

The evaluation of serum lysozyme activity followed the approach outlined by Demers and Bayne [38], which involves the lysis of the lysozyme-sensitive bacterium *Micrococcus luteus* (Sigma). A standard was established using hen egg white lysozyme (Sigma), with dilutions ranging from 0 to 20 *μ*L mL^−1^, prepared in a 0.1 M phosphate citrate buffer at pH 5.8. The standard, along with an undiluted serum sample (25 *μ*L), was then placed in triplicate within the wells of a 96-well plate. Subsequently, 175 *μ*L of *M. luteus* suspension (75 mg mL^−1^), prepared in the same buffer, was introduced into each well. Following rapid mixing, alterations in turbidity were gauged every 30 s over a 5-min span at 450 nm and approximately 20°C, utilizing a microplate reader. The measurement of alternative complement (ACH50) activity was conducted employing the methodology introduced by Sunyer and Tort [39], with adjustments as previously detailed by Yeh et al. [40]. The serum complement volume responsible for inducing 50% hemolysis (ACH50) was established, and the number of ACH50 U mL^−1^ was computed for the specimen. For serum total immunoglobulin (Ig) levels, precipitation of Ig using polyethylene glycol solution was employed as described by Siwicki and Anderson [41].

#### 2.4.4. Stress Parameters

Serum glucose levels were assessed through a commercially available kit (Pars Azmun, Karaj, Iran) in adherence to the manufacturer's protocol. The quantification of serum cortisol concentrations was performed using the competitive ELISA technique, utilizing a commercial kit (IBL Co., Gesellschaft für Immunchemie und Immunbiologie). For serum enzymatic activities including alkaline phosphatase (ALP), alanine aminotransferase (ALT), and aspartate transaminase (AST), commercial kits (Pars Azmun, Karaj, Iran) [42] were employed in conjunction with an automated biochemical analyzer (Beckman Coulter, USA).

#### 2.4.5. Antioxidant Enzyme Activities

Serum glutathione peroxidase (GPx) and superoxide dismutase (SOD) activities were assessed by evaluating the speed of glutathione oxidation and the pace of cytochrome C reduction, correspondingly. These evaluations were conducted using commercially available kits (ZellBio GmbH, Veltinerweg). The measurement of serum catalase activity was executed by determining the rate of hydrogen peroxide decomposition, following the methodology outlined by Goth [43]. Additionally, serum malondialdehyde (MDA) levels were determined using a commercial kit (ZellBio GmbH, Veltinerweg) based on the thiobarbituric acid technique.

### 2.5. Statistical Analysis

Initially, the conformity of the data to a normal distribution was assessed via the Shapiro–Wilk test. Subsequently, the homogeneity of variance was examined using Levene's test. Following this, distinctions among the groups were identified through a one-way analysis of variance (ANOVA), accompanied by Duncan's multiple range test, with a significance level set at *P*  < 0.05. Lastly, a two-way ANOVA was conducted to assess the impacts two factors: thermal stress and *P. abrotanoides* extract levels (PAE). All statistical analyses were carried out using SPSS software (version 22), and a significance level of *P*  < 0.05 was adopted as the threshold for acceptance.

## 3. Results

### 3.1. Growth and Feed Assimilation of Carp Juveniles Fed on *P. abrotanoides* Extract

**Figure 1:**
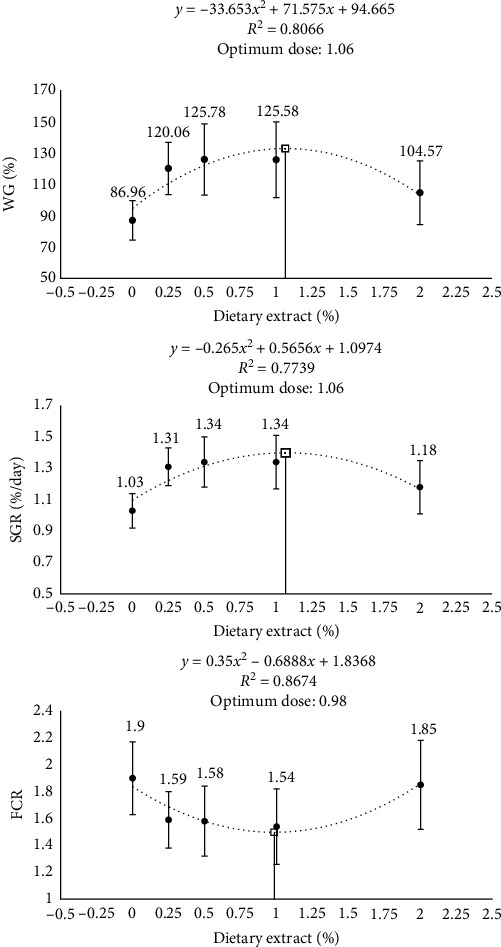
The relationship between weight gain (WG%), specific growth rate (%/day), and feed conversion ratio (FCR) against dietary *P. abrotanoides* extract levels (% diet) that are supplied in the diets of common carp for 60 days as described by second-order polynomial regression analysis.

Upon the conclusion of the 60-day feeding trial, the survival rate (%) of the fish was uniform across all experimental groups, with no instances of recorded mortality. Carp juveniles that were provided diets enriched with extracts (0.25%, 0.5%, 0.1%, and 2%) from *P. abrotanoides* for 60 days showed higher final weight (FW), weight gain (WG), and SGR than fish fed a control diet (*P*  < 0.001). Moreover, FCR improved significantly (displayed lower values) in fish-fed extract-supplemented diets (*P*=0.003). The highest FW, WG, and WG% were found in fish-fed diets supplemented with 0.5% and 1% *P. abrotanoides* extract, whereas the best FCR values were found in fish-fed diets supplemented with 1% and 0.5% extract.  Figure 1 illustrates the correlations between WG, SGR, and FCR of common carp concerning varying levels of dietary PAE. The second-order polynomial regression equations indicate an approximate optimal dietary PAE supplementation level of around 1% in fish diets.

Values of growth and feed assimilation of carp juveniles fed on *P. abrotanoides* extract are presented in Table 3.

### 3.2. Digestive Enzyme Activities

**Figure 2:**
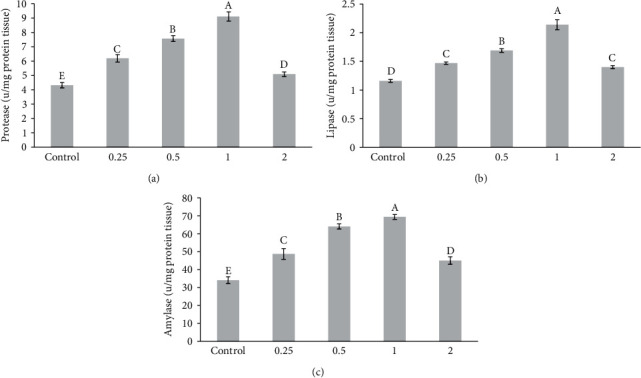
Intestinal digestive enzymes' activities in carp juveniles fed on *P. abrotanoides* extract after 60 days. Different letters above the bars show significant difference among the treatments (*n* = 3; Duncan test) (a–c).

All fish fed *P. abrotanoides* extract-enriched diets had significantly increased protease and lipase activities than control fish (Figures 2(a) and 2(b); *P*  < 0.001). The highest protease and lipase activity was found in fish-fed diets supplemented with 1% and 0.5% *P. abrotanoides* extract, whereas the lowest activity was seen in fish-fed diets supplemented with 2% extract. Amylase activity was much higher in fish-fed enriched diets containing 1% followed by 0.5% *P. abrotanoides* extract (Figure 2(c); *P*  < 0.001).

### 3.3. Serum Immune Parameters

The levels of all three immunological indicators examined in this study (ACH50, lysozyme, and total immunoglobulin) improved considerably when carps were exposed to *P. abrotanoides* extract for 60 days. The highest lysozyme activity was observed in fish that consumed a diet enriched with 1% extract, with the subsequent highest activity seen in those fed a diet containing 0.5% extract (*P*  < 0.001). While fish fed a 0.25% extract-enriched diet exhibited the lowest lysozyme activity. In addition, feed supplementation with 1% and 0.5% *P. abrotanoides* extract resulted in the highest total immunoglobulin levels (*P*  < 0.001). The highest levels of ACH50 were found in fish fed a diet supplemented with 0.5% followed by 1% extract (*P*=0.004). The serum immunological parameters of carp juveniles fed *P. abrotanoides* extract are shown in Table 4.

### 3.4. Serum Biochemical Parameters

The blood total protein and albumin levels of fish fed the *P. abrotanoides* extract for 60 days were considerably greater than those of fish fed the control diet. The highest levels of serum total protein were found in fish fed diets supplemented with 0.25% and 2% *P. abrotanoides* extract, respectively (*P*  < 0.001), whereas serum albumin levels were the highest in fish-fed diets supplemented with 2% and 1% *P. abrotanoides* extract, respectively (*P*  < 0.001). The serum total cholesterol levels in fish-fed diets containing 0.25% *P. abrotanoides* extract were significantly lower. A diet containing 2% extract, however, significantly increased serum total cholesterol levels when compared to a control diet (*P*  < 0.001). Fish that consumed diets containing 1% and 0.25% of *P. abrotanoides* extract exhibited the least triglyceride levels, respectively (*P*  < 0.001). Results of serum biochemical parameters of carp juveniles fed on *P. abrotanoides* extract are presented in Table 5.

### 3.5. Antioxidant Enzyme Activities before and after the Stress

The antioxidant status of fish was dramatically increased by supplementing their diet with *P. abrotanoides* extract. In contrast to the control group, the addition of 1% *P. abrotanoides* extract significantly elevated SOD levels, while 0.5% extract increased GPx activity (*P*  < 0.001). *P. abrotanoides* extract at 0.5% considerably reduced MDA levels, while 0.25% extract reduced catalase levels when compared to the control group (*P*  < 0.001). MDA, a lipid peroxidation biomarker, increased considerably in fish that were subjected to heat stress for 48 hr. The highest MDA levels were found in fish fed a control diet, while the lowest MDA levels were found in fish fed an enriched diet (0.5% and 1% *P. abrotanoides* extract). Catalase levels rose dramatically in fish exposed to heat stress, with fish fed a control diet having the highest levels, and fish provided an enhanced diet containing 1% *P. abrotanoides* extract having the lowest (*P*  < 0.001). In contrast to the control group, dietary addition of *P. abrotanoides* extract increased SOD and GPx activity following heat stress (*P*  < 0.001). Fish fed with 0.5% and 1% extract had the highest SOD activity. Furthermore, fish fed 1% extract had the highest GPx activity, followed by fish fed 0.5% extract.  Table 6 shows the antioxidant enzyme activities in carp juveniles fed *P. abrotanoides* extract.

### 3.6. Levels of Liver Enzymes, Glucose, and Cortisol before and after the Stress

In contrast to the control group, diet supplementation with various levels of *P. abrotanoides* extract for 60 days significantly reduced liver enzymes, glucose, and cortisol levels. The fish fed diet enriched with 1% *P. abrotanoides* extract had the lowest AST and ALT levels (*P*  < 0.001). The fish fed diet containing 0.25% extract exhibited the lowest ALP levels, followed by the diet enriched with 0.5% extract (*P*  < 0.001). Furthermore, the diet supplementation with 1% extract revealed the lowest glucose and cortisol levels (*P*  < 0.001). In contrast to the control group, carp juveniles exposed to heat stress and fed a diet enriched with *P. abrotanoides* extract had lower liver enzymes, glucose, and cortisol levels (*P*  < 0.001). Fish that were fed a diet enriched with 1% *P. abrotanoides* extract displayed the most minimal AST and ALT levels, while the lowest ALP levels were observed in fish fed with a diet supplemented with 0.25% followed by 0.5% extract, respectively. Diet supplementation with 1% extract showed the lowest glucose and cortisol levels when compared to the control group (*P*  < 0.001). Liver enzymes, glucose, and cortisol levels of carp juveniles fed on *P. abrotanoides* extract before and after stress are presented in Table 7.

## 4. Discussion

This preliminary study investigated the supplementation of *P. abrotanoides* extract (PAE) at 0.25%–2% and had a positive effect on overall performance, feed utilization, and digestive system of common carp. Effective metabolites, particularly from PAE, could be beneficial to the health status of the fish, which would result in a better growth performance. Moreover, the use of PAE promotes increased secretion of protease, lipase, and amylase enzymes in fish intestines, leading to more efficient nutrient absorption and better fish development. Similar to this study, the addition of herbal extracts to fish feeds increased digestive enzymes activities and growth performance of fish [44, 45, 46]. Moreover, the improvement of performance in common carp fed diets incorporated with PAE may be due to antioxidant exogenous enzymes. These enzymes may reduce to different stressors (physical stressors, environmental pollutants, chemotherapeutic drug applications, etc.) in fish production condition as with other plant extract applications [47, 48, 49]. Alamdari et al. [27] reported that leaf sample of *P. abrotanoides* contained the highest total phenolic compounds, flavonoids, and antioxidant activity by .17 ± 0.74 mg GAE/100 g dried sample weight, 2.26 ± 0.60 mg QE/g dried sample, and 69.10% ± 0.83%, respectively.

In fish, serum total protein is calculated as the sum of albumin and globulins. Increases in these proteins are considered an indicator of stronger immunity [50]. In this study, it was noticed that the highest serum total protein level was found in fish fed with 0.25% PAE group. Parallel with our study, increased serum protein values have been recorded in *C. carpio* fed with mix of *Malvae sylvestris*, *Origanum vulgare*, and *Allium hirtifolium* [51], *Pandanus tectorius* extract [52], and *Scrophularia striata* extract [53].

In our study, cholesterol and triglyceride levels in the serum decreased when fish was fed PAE supplemented diets, especially at level of 0.25%. Similarly, when herbal extracts like *Avena sativa* extract [54], dandelion extract [55], *Coriandrum sativum*, *Malva sylvestris* and *Quercus brantii* [56] were added to diets of *C. carpio*, it was reported that they decreased serum cholesterol or triglyceride levels of fish. These hypolipidemic effects of herbal extracts can be explained by the inhibiting the activity of HMG-CoA reductase in the liver and inhibiting intestinal acyl CoA: cholesterol acyl transferase and/or increases of the serum lipoprotein lipase and hepatic lipase activities as reported previously [57, 58, 59].

ACH50 and lysozyme are important parameters benefitted in the evaluation of innate immune system in fish. In this study, serum ACH50 and lysozyme activities significantly raised in PAE incorporated groups (0.5%–2%) compared to the control group before the stress exposure. Moreover, supplementation with PAE improved the immune system of fish in the present study, demonstrated by the enchanted total immunoglobulin concentration in the serum. Similarly, previous reports suggest that increasing ACH50, lysozyme, and/or total immunoglobulin activities in fish fed diets incorporated with exogenous herbal feed additives [48, 49, 60]. The increase in immune parameters of fish fed with PAE can be attributed to stronger antioxidant defenses, as a positive relationship between antioxidant enzyme activities, and immune responses has been reported in many studies before. In conclusion, the fish seem to be able to develop antioxidant defenses and produce more immune components when PAE is administered. According to the published report, a total of 26 components have been found in *P. abrotanoides* during the flowering stage. According to the findings, carotol (31.15%) was the most abundant ingredient in the *P. abrotanoides* extract, followed by naphthalene, 1,2,3,5,6,8a-hexahydro-4, 7-dimethyl-1-(1-methylethyl) (1S-cis) (14.251%), and agarospirol (7.233%). The composition of IR-alpha-pinene (0.268%) was likewise found to have the lowest value [27].

Antioxidant defense systems of aquatic organisms include free radical scavengers, reduced glutathione (GSH), and specific antioxidant enzymes such as catalase (CAT), superoxide dismutase (SOD), and glutathione peroxidase (GPx). The increased malondialdehyde (MDA) content is routinely used as a biomarker of lipid peroxidation in the liver [61]. In the present study, increases in SOD and GPx activities by dietary PAE before high-temperature exposure, which corresponds to the improved antioxidant capacity, are supported by lower MDA content. Similar results are in full agreement with findings of earlier studies in common carp, fed different herbal extracts [62, 63, 64]. Moreover, previous studies showed that antioxidant responses exhibit a vital role in antioxidant response in different stress conditions like high temperature [30, 65], ammonia stress [10], high stocking density and/or acute crowding stress [66, 67], hypoxia [65], and cold temperature [68]. As a result, the better antioxidant status following incorporations with PAE observed in this study may be indicative of higher stress prevention capacity in fish. Similarly, oral administration of anthraquinone extract supplement have resulted increased SOD and decreased MDA levels in giant freshwater prawn, *Macrobrachium rosenbergii*, under high-temperature stress [69].

Cortisol is used as a main stress indicator in fish studies, while serum glucose is a subsequent one [70]. In this study, high-temperature exposure elevated cortisol and glucose levels in fish serum. Increase in serum cortisol level is response to adrenocorticotropic hormone (ACTH) which is the dominant secretagogue of cortisol release from inter-renal tissue under stress conditions [71, 72]. Additionally, high levels of glucose in common carp serum under high-temperature stress may be a result of increased energy requirements during stress [73, 74]. A similar increase in serum glucose and cortisol levels at higher temperature was observed in Wuchang bream, *Megalobrama amblycephala* [73]. In contrast to our findings, however, reductions in glucose level in rainbow trout serum was observed under higher temperature (21°C) stress conditions [65]. These different results might be associated with differences in fish species, fish size, fish age, rearing conditions, and exposure time.

Previous studies reported that herbal extracts showed a remarkable antistress effect on fish [24]. The role of herbal extracts is probably attributed to the effect of active metabolites in improving the oxidative condition and organ health. Based on the present results, when fish were administered with 0.25%–1% PAE diets, the cortisol and glucose levels in fish serum significantly decreased in the poststress groups. This was supported in a recent study, where anthraquinone extract from *R. officinale* Bail decrease heat-induced serum glucose and/or cortisol elevation in fish [73].

Serum enzyme variables are accepted important indexes of tissue damages; for example, AST and ALT are indexes of liver damage [75] and ALP for biliary tract damages in fish [76]. Therefore, enhances in AST, ALT, and/or ALP in fish serum could be indicator for liver damage. In this study, serum AST, ALT, and ALP levels were markedly decreased when the fish were fed with different PAE levels before and after high-temperature stress. Previous reports showed that serum AST, ALT, and/or ALP activities provided a decrease when common carps were fed with herbal extracts [51, 77]. To our knowledge, no studies investigated the serum enzyme levels of fish fed with PAE-supplemented feeds were found under the high-temperature stress. Liu et al. [73] reported that AST and ALT activities in Wuchang bream, *M*. *amblycephala*, fed a diet containing anthraquinone extract under high-temperature stress were significantly lower than those of the control fish. Moreover, Liu et al. [69] reported that dietary anthraquinone extract removed the adverse effects of high-temperature stress on AST and ALT levels in serum in the freshwater prawn, *M. rosenbergii*.

## 5. Conclusions

In summary, the findings of this study indicate that including *Perovskia abrotanoides* extract (PAE) in the diets significantly enhanced growth performance, bolstered digestive enzymes, and improved immune parameters and antioxidant status in common carp while also providing protection against high-temperature fish stress.

## Figures and Tables

**Table 1:** GC-MS analysis of aerial parts of *P abrotanoides* at flowering stage.

Row	Name of the compound	Composition percentage	Retention time
1	IR-alpha-pinene	0.268	3.302
2	Eucalyptol	1.396	4.705
3	Bicyclo(2,2,1)heptan-2-one, 1,7,7-trimethyl (1S)	1.024	7.040
4	Bornyl acetate	1.654	10.026
5	Cyclohexene-1-methanol,alpha-4-trimethyl-propanoate	3.395	11.537
6	Copaene	0.779	12.208
7	1H-Cycloprop(e)azulene,1a.2.3,4,4a,5,6,7b-octahydro-1,1,4,7-tetramethyl-(1 a R1a alpha .4.alpha, 4 a. beta, 7 balpha)	0.952	12.981
8	Caryophyllene	2.370	13.343
9	Azulene,1,2,3,4,5,6,7,8-octahydro1,4-dimethyl-7-(1-methyle)-(1S)-(1.alpha,4.alpha,7.alpha)	2.220	14.213
10	1H-Cycloprop(e)azulene,decahdro-1,1,7-trimethyl-4-methylene(1a, alpha,4 a.beta,7 alpha,7 beta, 7 b. alpha)	0.788	14.306
11	Naphthalene,1,2, 3, 4,4a,5,6,8a-octahydro-7-methyl-4-methylene-1-1 methylethyl)-(1 alpha,4 a beta, 8 a alpha)	2.858	15.570
12	Naphthalene,1,2,3,5,6,8-a-hexahydro 4,7-dimethyl-1,(1-methylethy) (1S-cis)	3.736	15.655
13	2,3,4-Trifluorobenzoic acid,2,4,6 trichlorophenyl ester	1.908	15.773
14	Isoaromadendrene epoxide	1.473	17.279
15	Aristolene epoxide	1.720	17.936
16	Cubenol	1.597	18.013
17	Beta-Guaiene	1.204	18.276
18	1-Naphthalenol,decahydro-1,4a-dimethyl-7,(1-methylethylid) 1 R-(1.alpha,4a.beta,8a.alpha)	3.644	18.575
19	Naphthalene,1,2,3,5,6,8a-hexahydro-4,7-dimethyl-1-(1-methylethyl)(1S-cis)	14.251	18.773
20	Patchoulene	5.516	18.847
21	Ir,4s,7s.11R-2,2,4,8 -Tetramethyltricyclo (5,3,1,0 (4,11))undec-8ene	1.805	19.042
22	Agarospirol	7.233	19.419
23	1H-Cyclopropeazulene,1a,2,3,4,4a,5,6,7b-octahydro-1,1,4,7-tetramethyl-(1a.alpha.4alpha,4a.beta),7b.alpha	2.865	19.765
24	Carotol	**31.105**	**20.009**
25	7-1-Isopropyl-1,1,4a,trimethyl,1,2,3,4,4a,9,10,10a-octahydrophenanthrene	0.904	26.918
26	1R,4s,7s,11R-2,2,4,8-tetramethyl tricyclo(5, 3, 1,0 (4,1))undec-8-ene	1.017	27.511

Bold values indicate more of these compounds in the extract.

**Table 2:** Dietary formulation and proximate composition analysis of experimental diets (% on dry matter basis) containing different levels of phenolic compounds of *P. abrotanoides* extract.

Ingredients (%)	Experimental diets				
Fishmeal^1^	10	10	10	10	10
Meat meal^2^	20	20	20	20	20
Soybean meal	23	23	23	23	23
Wheat meal	34.9	34.65	34.4	33.9	32.9
Fish oil	0.7	0.7	0.7	0.7	0.7
Soybean oil	0.7	0.7	0.7	0.7	0.7
Corn flour	9	9	9	9	9
L-Lysine^3^	0.7	0.7	0.7	0.7	0.7
L-Methionine 100^3^	0.5	0.5	0.5	0.5	0.5
Vitamin premix^a^	0.25	0.25	0.25	0.25	0.25
Mineral premix^b^	0.25	0.25	0.25	0.25	0.25

*Perovskia* extract	0	0.25	0.5	1	2

Dry matter	89.18	89.15	89.13	89.09	89.00
Crude protein (%)	35.03	35.03	35.03	35.02	35.00
Crude fat (%)	5.78	5.78	5.78	5.78	5.78
Crude ash (%)	5.82	5.82	5.82	5.82	5.82
Energy (kcal/kg)	4,052.99	4,052.01	4,051.03	4,049.07	4,045.15

^1^Pars Kilka Co., Mazandaran, Iran (Kilka powder analysis; protein, 70%–72%; fat, 8%–11%; ash, 11.6%; moisture, 7%–9%). ^2^Makianmehr Co., Golestan, Iran. ^3^Morghenojan.Co., Tehran, Iran. ^a^Vitamin premix (per kg of diet): vitamin A, 2,000 IU; vitamin B_1_ (thiamin), 5 mg; vitamin B_2_ (riboflavin), 5 mg; vitamin B_6_, 5 mg; vitamin B_12_, 0.025 mg; vitamin D_3_, 1,200 IU; vitamin E, 63 mg; vitamin K_3_, 2.5 mg; folic acid, 1.3 mg; biotin, 0.05 mg; pantothenic acid calcium, 20 mg; inositol, 60 mg; ascorbic acid (35%), 110 mg; niacinamide, 25 mg. ^b^Mineral premix (per kg of diet): MnSO_4_, 10 mg; MgSO_4_, 10 mg; KCl, 95 mg; NaCl, 165 mg; ZnSO_4_, 20 mg; KI, 1 mg; CuSO_4_, 12.5 mg; FeSO_4_, 105 mg; Co, 1.5 mg.

**Table 3:** Growth performance and feed efficiency of common carp fed with *P. abrotanoides* extract after 60 days.

	Control	0.25	0.5	1	2	*P-value*
IW (gr)	18.17 ± 0.71	17.74 ± 0.88	17.55 ± 0.96	17.50 ± 0.89	18.02 ± 0.86	*P* > 0.001
FW (gr)	33.97 ± 2.50c	38.99 ± 2.79ab	39.51 ± 3.31a	39.36 ± 3.33a	36.73 ± 2.47b	*P* < 0.001
WG (gr)	15.79 ± 2.29c	21.24 ± 2.72a	21.96 ± 3.45a	21.85 ± 3.61a	18.71 ± 3.00b	*P* < 0.001
WG (%)	86.96 ± 12.61c	120.06 ± 16.66ab	125.78 ± 22.74a	125.58 ± 24.09a	104.57 ± 20.28b	*P* < 0.001
SGR (%/d)	1.03 ± 0.11c	1.31 ± 0.12ab	1.34 ± 0.16a	1.34 ± 0.17a	1.18 ± 0.17b	*P* < 0.001
FCR	1.90 ± 0.27a	1.59 ± 0.21b	1.58 ± 0.26b	1.54 ± 0.28b	1.85 ± 0.33a	*P*=0.003
SR (%)	100	100	100	100	100	*P* > 0.001

Different letters within a row indicate significant differences among the treatments (*n* = 3; Duncan test).

**Table 4:** Serum immunological parameters of carp juveniles fed *P. abrotanoides* extract after 60 days.

Experiment groups	Parameters		
	ACH50 (u/mL)	Lysozyme activity (u/mL/min)	Total immunoglobulin (mg/mL)
0	134.66 ± 0.49^c^	22.28 ± 0.34^c^	17.03 ± 0.60^b^
0.25	136.21 ± 1.09^b^	22.60 ± 0.81^c^	17.11 ± 0.54^b^
0.5	137.89 ± 1.14^a^	26.76 ± 1.87^ab^	18.88 ± 0.31^a^
1.0	137.54 ± 0.58^ab^	28.50 ± 0.63^a^	18.90 ± 0.20^a^
2.0	136.14 ± 0.32^b^	25.89 ± 0.53^b^	18.16 ± 0.24^a^
*P-value*	*P*=0.004	*P* < 0.001	*P* < 0.001

Different letters within a column indicate significant differences among the treatments (*n* = 3; Duncan test).

**Table 5:** Serum biochemical parameters of carp juveniles fed on *P. abrotanoides* extract after 60 days.

Experiment groups	Parameters				
	Total protein (gr/dL)	Albumin (gr/dL)	Globulin (gr/dL)	Cholesterol (mg/dL)	Triglycerides (mg/dL)
0	3.07 ± 0.12^d^	1.35 ± 0.036^c^	1.72 ± 0.11^b^	165.92 ± 2.89^b^	150.24 ± 2.75^a^
0.25	3.57 ± 0.08^a^	1.37 ± 0.026^c^	2.2 ± 0.06^a^	141.76 ± 3.06^c^	135.04 ± 1.37^b^
0.5	3.21 ± 0.06^cd^	1.37 ± 0.026^c^	1.84 ± 0.078^b^	159.74 ± 1.23^b^	146.43 ± 4.39^a^
1.0	3.25 ± 0.05^bc^	1.45 ± 0.020^b^	1.8 ± 0.036^b^	160.66 ± 4.72^b^	119.55 ± 0.99^c^
2.0	3.37 ± 0.04^b^	1.57 ± 0.010^a^	1.8 ± 0.045^b^	182.67 ± 3.95^a^	150.26 ± 1.31^a^
*P-value*	*P* < 0.001	*P* < 0.001	*P* < 0.001	*P* < 0.001	*P* < 0.001

Different letters within a column indicate significant differences among the treatments (*n* = 3; Duncan test).

**Table 6:** Serum antioxidant enzyme activities (U mg^−1^ protein) andoxidative status (nmol MDA mg^−1^ protein) of common carp fed *P. abrotanoides* extract before and after high-temperature stress (32°C).

Experiment groups	Parameters			
	SOD (U/mL)	GPX (U/mL)	Catalase (U/mL)	MDA (*µ*mol/L)
Before stress				
0	82.33 ± 1.32^d^D	139.09 ± 2.13^d^G	122.14 ± 3.34^a^C	143.72 ± 5.69^a^B
0.25	87.25 ± 0.90^c^C	149.32 ± 1.22^c^F	88.32 ± 3.10^d^F	88.41 ± 2.62^b^F
0.5	89.81 ± 0.71^b^B	190.66 ± 2.71^a^C	100.38 ± 3.78^c^E	76.54 ± 2.43^c^G
1.0	91.57 ± 0.53^a^B	171.43 ± 1.36^b^D	90.51 ± 2.06^d^F	88.61 ± 5.26^b^F
2.0	87.34 ± 0.49^c^C	140.37 ± 2.43^d^G	111.58 ± 2.81^b^D	90.87 ± 5.96^b^F
After stress				
0	70.91 ± 1.81^d^E	124.88 ± 5.09^e^H	163.08 ± 3.10^a^A	179.31 ± 3.48^a^A
0.25	85.60 ± 1.04^b^C	160.94 ± 5.66^d^E	121.21 ± 2.72^c^C	120.11 ± 1.89^c^D
0.5	97.26 ± 1.06^a^A	201.09 ± 6.95^b^B	123.88 ± 7.02^c^C	102.47 ± 3.92^d^E
1.0	97.20 ± 0.60^a^A	215.40 ± 3.05^a^A	108.13 ± 6.82^d^D	102.28 ± 2.09^d^E
2.0	81.92 ± 1.23^c^D	171.33 ± 6.50^c^D	142.33 ± 2.46^b^B	129.86 ± 3.10^b^C
Two-way ANOVA				
Stress	*P*=0.010	*P* < 0.001	*P* < 0.001	*P* < 0.001
Diet	*P* < 0.001	*P* < 0.001	*P* < 0.001	*P* < 0.001
Interaction	*P* < 0.001	*P* < 0.001	*P*=0.001	*P* < 0.001

Different letters within a column indicate significant differences among the treatment (*n* = 3; Duncan test).

**Table 7:** Stress indicators of common carp fed *P. abrotanoides* extract before and after high-temperature stress(32°C).

Experiment groups	Parameters				
	Cortisol (ng/mL)	Glucose (mg/dL)	AST (u/L)	ALT (u/L)	ALP (u/L)
Before stress					
0	189.74 ± 3.99^a^C	97.45 ± 0.93^a^BC	131.62 ± 3.89^a^CD	28.39 ± 0.60^a^D	140.79 ± 5.03^a^B
0.25	149.41 ± 5.94^b^E	84.15 ± 2.77^b^D	113.10 ± 1.56^b^F	23.60 ± 0.72^b^E	111.01 ± 4.22^c^F
0.5	118.51 ± 5.18^c^G	83.82 ± 4.43^b^D	107.25 ± 2.23^c^FG	21.42 ± 0.65cF	113.22 ± 7.09^c^EF
1.0	111.50 ± 3.41^c^G	72.81 ± 4.50^c^E	102.36 ± 2.62^d^G	20.29 ± 0.66^c^F	119.26 ± 2.65^bc^DEF
2.0	183.32 ± 3.62^a^C	96.92 ± 1.92^a^BC	115.76 ± 1.45^b^EF	21.42 ± 0.83^c^F	124.56 ± 2.95^b^CD
After stress					
0	245.42 ± 4.75^a^A	138.97 ± 3.59^a^A	168.93 ± 3.40^a^A	45.89 ± 1.51^a^A	150.15 ± 3.87^a^A
0.25	170.09 ± 8.06^c^D	101.72 ± 7.18^b^B	138.33 ± 7.14^bc^BC	35.03 ± 0.76^bc^BC	111.48 ± 3.30^c^F
0.5	137.43 ± 4.35^d^F	96.24 ± 3.37^b^BC	126.89 ± 3.89^cd^D	33.31 ± 0.68^c^C	115.50 ± 4.77^c^EF
1.0	135.90 ± 5.82^d^F	94.17 ± 3.50^b^C	122.63 ± 8.54^d^DE	29.62 ± 1.55^d^D	120.48 ± 4.07^c^DE
2.0	230.37 ± 6.42^b^B	132.85 ± 4.04^a^A	143.92 ± 8.66^b^B	36.48 ± 1.47^b^B	129.84 ± 7.27^b^C
Two-way ANOVA					
Stress	*P* < 0.001	*P* < 0.001	*P* < 0.001	*P* < 0.001	*P*=0.045
Diet	*P* < 0.001	*P* < 0.001	*P* < 0.001	*P* < 0.001	*P* < 0.001
Interaction	*P* < 0.001	*P* < 0.001	*P*=0.043	*P* < 0.001	NS

Different letters within a column indicate significant differences among the treatment (*n* = 3; Duncan test).

## Data Availability

The data are available from the corresponding author upon reasonable request.
